# Steroid Cell Tumour in Pregnancy: Reflection on a Rare Case and Review of the Literature

**DOI:** 10.1155/2020/1817042

**Published:** 2020-02-28

**Authors:** J. Weishaupt, U. Herbst

**Affiliations:** Department of Gynaecology Oncology, Liverpool Cancer Therapy Centre, Liverpool Hospital, Liverpool, NSW, Australia 2170

## Abstract

There are only a few cases of steroid cell tumours that have been described in the literature. Here, we present an exceptionally rare case of a steroid cell tumour arising from the ovary in early pregnancy.

## 1. Introduction

Steroid cell tumours, which used to be called lipid cell tumours, are rare sex cord tumours. They account for approximately 0.1% of all ovarian tumours. These tumours are subdivided into 3 types: stromal luteoma, Leydig cell tumours, and steroid cell tumours not otherwise specified (NOS). Steroid cell tumours NOS are the most common subtype accounting for about 60% of these tumours. First described by Scully et al., they cannot be categorized as either stromal luteoma or Leydig cell tumours and should be considered a diagnosis of exclusion as their significance is that the cell lineage is not defined [1]. In pregnancy, these tumours are exceptionally rare and must be differentiated from luteoma of pregnancy and other ovarian malignancies. Steroid cell tumours usually develop in adults with the median age being 42. They characteristically present as a solid, well-circumscribed tumour and occasionally, as cystic tumours. They are almost always unilateral and are clinically malignant in up to a third of cases [1]. Clinically, 60% of these tumours show virilisation or androgenic changes and may be associated with oestrogen secretion in 6-23% of cases and may also present as Cushing syndrome.

## 2. Case Report

**Figure 1:**
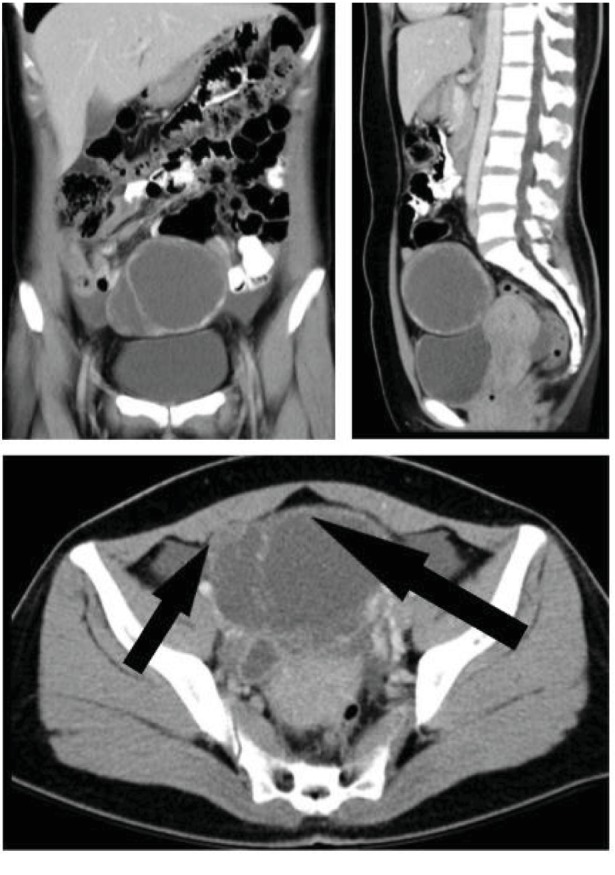
CT scan abdomen/pelvis shows a left adnexal, complex cystic lesion with enhancing, solid nodules along its wall (black arrows), likely arising from the left ovary.

Here, we present the case of a steroid cell tumour in pregnancy. A 32-year-old, Vietnamese female, gravida 2, para 1, who had one previous uncomplicated normal vaginal delivery nine years ago, presented at nine weeks of gestation for antenatal care in a tertiary obstetric unit in Sydney, Australia. Her only clinical symptom was occasional left-sided, lower abdominal pain. She had no features of virilisation and had no prior gynaecological history. Her clinical examination was unremarkable. She had recently moved to Australia from Vietnam, six months prior to her pregnancy, where an initial ultrasound demonstrated an ovarian mass. An abdominal/pelvic CT scan ordered by her general practitioner, unbeknown to a very early pregnancy, showed a left ovarian cyst, 100  × 80  × 80 mm in size, with solid nodules along the cyst wall, no ascites, a normal right ovary, and no lymphadenopathy or peritoneal enhancement (Figure 1). The Ca 125 was 265 kU/L at the initial consultation.

**Figure 2:**
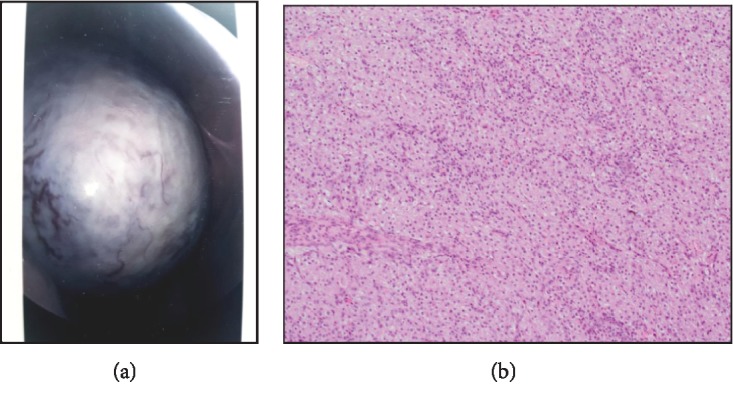
(a) Diagnostic laparoscopy: left adnexa consisted of a fallopian tube attached to a 120 × 120 × 100 mm grey, solid ovarian mass with a smooth surface and dilated vessels. (b) Microscopic appearance of a steroid cell tumour showing large arrogates of typical polygonal-to-round tumour cells having distinct cell borders, central nuclei, prominent nucleoli, and moderate-to-abundant spongy or eosinophilic granular cytoplasm, intersected by delicate fibrous bands (courtesy of Dr. Kasim Ismail, reporting pathologist, and Dr. Julia Low, Liverpool Hospital, NSW, Australia).

The gestational dating ultrasound confirmed a 102  × 94  × 71 mm well-circumscribed, left ovarian mass with an irregular, solid periphery and a large central cystic component measuring 82  × 62  × 52 mm associated with only a small amount of free pelvic fluid and no vascularity. The consensus was to perform a laparoscopic cystectomy, ideally in her second trimester by the gynaecology oncology team. She, however, presented to the emergency department at 14 weeks of gestation with rapidly accumulating ascites and associated worsening abdominal pain, and her Ca 125 had increased to 1700 kU/L. An ultrasound-guided ascitic drainage identified no malignant cells and 1000 mL of fluid was drained. A laparoscopy was performed at 15 weeks, and upon inspection of the left adnexa, a grey, complex mass measuring 70 × 100 mm was noted to be consuming the left ovary. The intraoperative decision to convert to a midline laparotomy to avoid surgical spillage was made (Figure 2(a)). A left salpingo-oophorectomy and omental biopsy were performed. The mass was removed without spillage. The right adnexa appeared normal. The frozen section demonstrated a solid/cystic stromal lesion, and an epithelial component could not be excluded.

Her postoperative recovery was uneventful, and she was referred to the high-risk antenatal clinic for ongoing perinatal care. At 38 weeks of gestation, she went into spontaneous labour and delivered a healthy 3 kg baby boy with a normal Apgar score. She will have gynaecology oncology follow-up post delivery.

## 3. Pathology

The sectioned surface of the left ovary on gross examination was smooth and grey with a solid, multinodular, and cystic appearance with focal haemorrhage and a thickened cyst wall of 10-20 mm. The three nodules described: a 25 × 20 × 5 mm, pale, yellow nodular area, underneath being a cystic cavity 50 × 30 × 25 mm filled with clear watery fluid, surrounded by yellowish nodules at the periphery 4-10 mm across. There was also a defined yellow, grey nodule 30 × 20 × 15 mm. The omentum and fallopian tube were normal.

On microscopic examination, the paraffin sections confirmed the frozen section appearance of a solid and cystic expansion of the left ovary, with a thin attenuated rim of ovarian stroma wrapped around the lesion. Nodules of viable epithelioid tumour cells blended seamlessly into the surrounding oedematous stroma, including the large areas of tumour necrosis. The constituent cells were large and seemingly arranged in small nests with a delicate vascular supportive framework. The nuclei were round with vesicular chromatin, prominent nucleoli, irregular nuclear membranes, occasional apoptosis, and simple mitoses were seen. The cytoplasm was abundant, vaguely granular/bubbly, and eosinophilic (Figure 2(b)). No Reinke crystals were seen, and PASD stain for mucin was negative. The immunostains showed most cells to be positive for calretinin, inhibin and synaptophysin while there was moderate positivity for vimentin and Melan-A. Very focal patchy staining was observed for Cam5.2. Nonspecific staining for PLAP was observed. S100, HMB-45, CD117, chromogranin, Oct3/4, HPL, Epithelial Membrane Antigen (EMA), PAX-8, and cytokeratins MNF-116 plus AE1/AE3 were negative. CD34 highlights the supportive vascular framework. The Ki-67 proliferation index is ~10%.

## 4. Discussion

An ovarian tumour presenting during pregnancy has an incidence ranging from 1 : 815 to 1 : 2200 (Table 1). Among these, the incidence of malignancy ranges from 2-8% with sex cord tumours, granulosa theca tumour, Krukenberg tumour, papillary mucinous cystadenocarcinoma, and mucinous cystadenocarcinoma are more commonly seen during pregnancy [2]. Steroid cell tumours of the ovary are especially rare in pregnancy with only a handful of case reports being described worldwide. With the increased use of ultrasound during pregnancy, findings of adnexal masses have become more frequent, and navigating the management of these masses during pregnancy can be challenging. Adnexal masses during pregnancy which increase the risk of malignancy should be surgically evaluated. These include a cystic mass (>5 cm) that is symptomatic and does not diminish in size or a solid, unilateral mass and ascites [3, 4]. Steroid cell tumours are mainly unilateral, and only 6% of patients have bilateral tumours. About 25% show no hormonal manifestations and are symptomatic with pain, as was the initial presentation in our patient [5].

As in this case, steroid cell tumours should primarily be managed surgically to preserve fertility and the fetus in the second trimester [3]. Steroid cell tumours have the potential to rupture or tort preoperatively particularly in pregnancy compared with nonpregnant women [6]. Explorative laparotomies with unilateral salpingo-oophorectomy and staging in women without evidence of malignancy on histopathology have excellent surgical outcomes despite no well-established surgical protocols. As they are uncommonly bilateral, a bilateral salpingo-oophorectomy is not indicated. For women who have completed their family, a total abdominal hysterectomy and bilateral salpingo-oophorectomy and complete surgical staging are recommended [5].

Most of these tumours are diagnosed early. There is limited data on the recurrence rate and little consensus on adjuvant therapy for advanced disease. Treatment should be based on the tumour histology, stage, and desire for fertility [6]. The most important factor to be determined in steroid cell tumours of the ovary is whether the tumour has malignant features or not. Fortunately, our patient's tumour was benign and did not exhibit any histological predictors of malignant behaviour. Hayes and Scully found that the most accurate predicator of malignancy was >2 mitotic figures per 10 high-power fields. Other features included grade 2-3 atypia, necrosis, haemorrhage, and a diameter of >7 cm [7].

When pathologists encounter a rare tumour such as this, they should use all the clinical, histopathological, immunohistochemical, and microscopic evidence available to determine its origin (Table 2). When this is unsuccessful, as in this case, they are designated steroid cell tumours NOS. Furthermore, the tumour in our case report was unilateral and presented prior to pregnancy which excluded a pregnancy luteoma. A pregnancy luteoma is often bilateral and resolves spontaneously after pregnancy [2]. There are no well-established protocols for managing this rare tumour together with the challenge of managing adnexal masses in pregnancy. A careful correlation between clinical evaluation, surgical evaluation, and microscopic analysis is necessary, as is regular follow-up.

## Figures and Tables

**Table 1:** Characteristics of sex cord-stroma tumours and luteoma in pregnancy [8–11].

Ovarian pathology	Bilateral (%)	Virilisation Maternal (%) (M), fetal (%) (F)	Malignancy (%)	Solid/cystic	Pregnancy symptoms	Pregnancy treatment
(A) Pregnancy luteoma	30-47	M = 25 − 35 F = 20	0	Solid	None/torsion/rupture	None/biopsy/self-limiting
(B) Sex cord-stroma tumours (histology (%) seen in pregnancy)	Variable	M = variable F = 0	The majority = stage I (76.1%)	Tumour size < 15 cm (64.9%)	^∗^Abdominal-pelvic pain (45.7%), ^∗^Palpable mass (30.4%), ^∗^Virilization (26.1%), ^∗^Torsion/rupture	Unilateral salpingo-oophorectomy (80.4%)
(1) Granulosa (22%)	5	M = 75 F = 0	5-25	Solid/cystic		
(2) Thecoma (18.5%)	10	F = 0	0	Solid		
(3) Sertoli-Leydig (8.5%)	5	M = 69‐87 F = 0	44	Solid		
(4) Hilar cell	<10	—	<10	—		
(5) Thecoma lutein cysts	96	M = 30‐50	0	Cystic		
(6) Stromal hyperthecosis	>50	0	0	—		
(7) Steroid cell	6	M = 56‐77	25-43	Solid/cystic		

**Table 2:** Clinical, histopathological, and immunohistochemistry (IHC) comparisons between steroid cell tumour NOS, pregnancy luteoma, and our case study [12–14].

	Steroid cell tumour, NOS	Pregnancy luteoma	Our case
Malignancy features (Hayes and Scully [12])	30%	Benign	Benign
(1) Tumour diameter > 7 cm	Yes	No	No
(2) Mitotic figures per 10 high-power fields ≥ 2	Yes	No	No
(3) Necrosis	Yes	No	Yes (due to torsion)
(4) Haemorrhage	Yes	No	Focal
(5) Grade 2/3 nuclear atypia	Yes	No (mild)	No (mild)

Reinke crystals	No	No	No

Tumour histopathology [12–14]	^∗^Macroscopically yellow ^∗^Diffusely arranged cells, also nests, clusters, cords, and columns ^∗^Tumour cells are round/polygonal with spongy to granular, eosinophilic cytoplasm with clear intracytoplasmic vacuoles ^∗^Distinct cell borders, central nuclei, prominent nucleoli	^∗^Macroscopically brown ^∗^Sharply circumscribed, rounded masses of polygonal cells with abundant pink cytoplasm, round nuclei, and variably prominent nucleoli	^∗^Macroscopically grey, with multinodular yellow components ^∗^Nodules of viable epithelioid tumour cells blend into the surrounding stroma, including areas of tumour necrosis ^∗^Cells arranged in small nests with a delicate vascular supportive framework ^∗^The cytoplasm is abundant with prominent round nuclei and irregular membranes

Unilateral/single	Yes (6% bilateral)	Bilateral in 1/2 and multiple 2/3	Yes

Lipids/stroma	Yes/scant stroma	Lipid-free steroid cells/scant stroma	Yes
IHC stains + VE [8, 9, 10]	Inhibin, fat stains (75%), vimentin (75%), Cam5.2 (46%), AE1/AE3 (37%), EMA (8%), and S100 (7%)	Alpha inhibin, cytokeratin, vimentin, CD 99	Calretinin, inhibin, synaptophysin, vimentin, and Melan-A

Diagnosis/treatment	Salpingo-oophorectomy	Biopsy/simple excision/no treatment	Salpingo-oophorectomy

Reoccurrence	None after oophorectomy	Regress after pregnancy	Limited data

Pregnancy	Rare	3^rd^ trimester—usually incidental	Diagnosed prepregnancy

Virilisation	2/3	1/4	No
